# Evolution of hermaphroditism decreases efficacy of Ascaroside#8-mediated mate attraction in *Caenorhabditis* nematodes

**DOI:** 10.17912/micropub.biology.000134

**Published:** 2019-07-19

**Authors:** Douglas K. Reilly, Lily J. Randle, Jagan Srinivasan

**Affiliations:** 1 Department of Biology and Biotechnology, Worcester Polytechnic Institute, Worcester, MA; 2 Department of Chemistry and Biochemistry, Worcester Polytechnic Institute, Worcester, MA; 3 Current Address: SBH Sciences, Natick, MA

**Figure 1. Caenorhabditis species males respond differentially to ascr#8:**
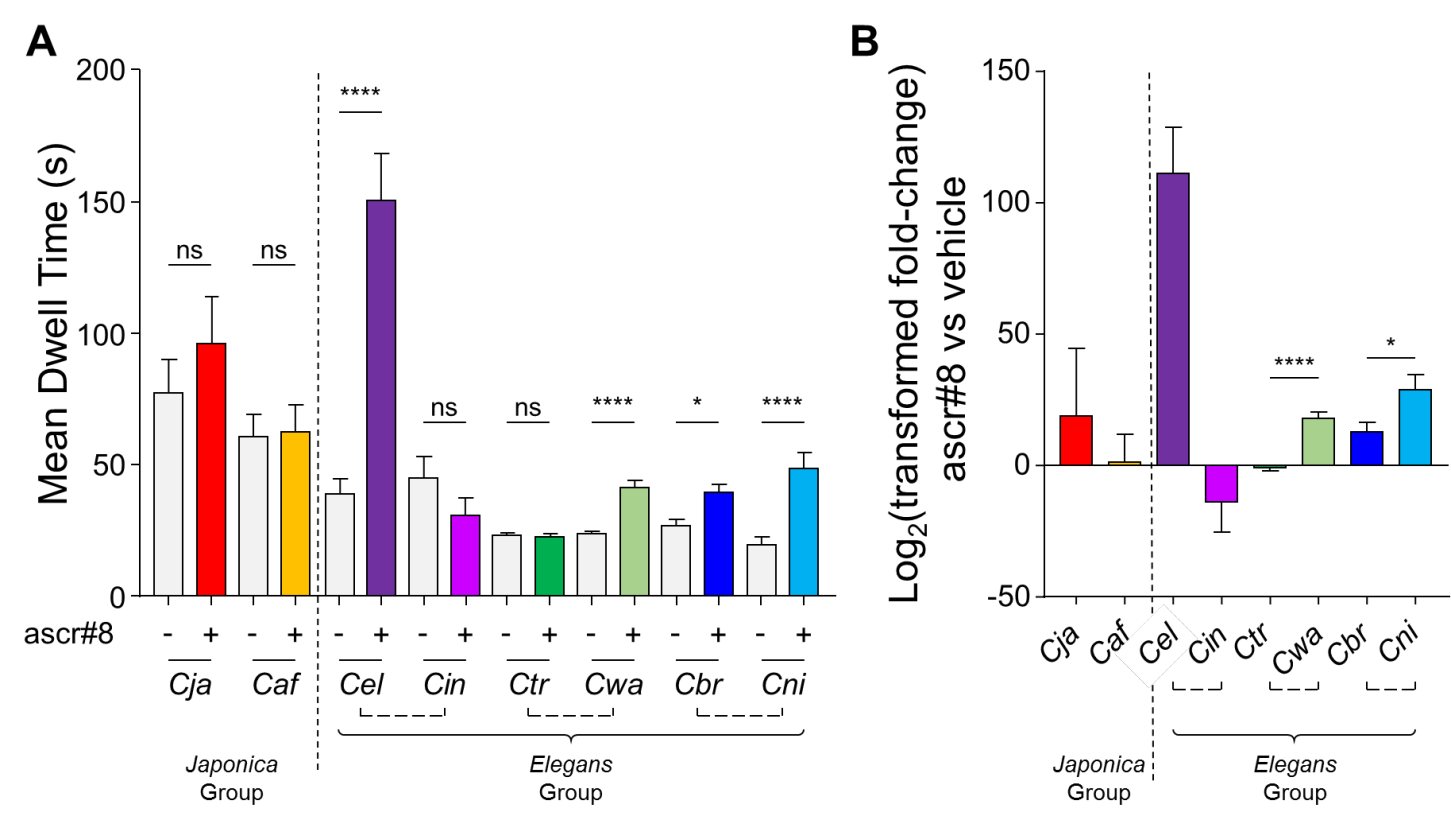
**(A)** Dwell times of *Caenorhabditis* males in vehicle control (-) and ascr#8 (+), respectively. Hermaphroditic species were tested alongside their gonochoristic sister-species (denoted by dotted horizontal brackets: *C. elegans* vs. *C. inopinata, C. briggsae* vs. *C. nigoni*, *C. tropicalis* vs. *C. wallacei*). The *Japonica* Group species, *C. japonica* and *C. afra*, (left of vertical dotted line) were also tested for their attractive response. Species are displayed according to the most recent phylogenetic analysis of the *Caenorhabditis* genus (Stevens *et al.*, 2019). No species were as attracted to ascr#8 as *C. elegans*, although *C. briggsae*, *C. nigoni*, and *C. wallacei* did spend significantly more time in ascr#8 compared to the vehicle. Error bars denote SEM. n ≥ 12. Paired t-tests or Wilcoxon test (dependent on Normality test) of ascr#8 vs. vehicle control. **(B)** The Log_2 _of Transformed fold-change of ascr#8 vs vehicle dwell times. See *Description* for transformation calculations. Among the sister-species pairs, gonochoristic species exhibited a significant increase in ascaroside dwell time. Student’s t-test. Brackets linking species denote sister-species pairs, with androdioecious species on the left, gonochoristic on the right. * *p*< 0.05, **** *p*< 0.0001.

## Description

Nematodes, such as the model organism *Caenorhabditis elegans*, communicate environmental and developmental information with conspecifics through a class of small-molecule pheromones termed ascarosides (Butcher, 2017; Chute and Srinivasan, 2014; Ludewig and Schroeder, 2013). Nematodes share ascaroside signaling pathways (Choe *et al.*, 2012), but are also capable of eavesdropping on chemical signals of predatory species (Liu *et al.*, 2018). Ascarosides signal vast arrays of information, either individually or as blends, based on concentration, sex, physiological state, and other ascarosides sensed (McGrath and Ruvinsky, 2019; Pungaliya *et al.*, 2009; Srinivasan *et al.*, 2008; Srinivasan *et al.*, 2012). For instance, octopamine-succinylated ascaroside #9 (osas#9) is able to signal starvation conditions in the absence of other ascarosides (Artyukhin *et al.*, 2013).

*C. elegans* (*Cel*) is an androdioecious species, with the majority of the natural population comprised of self-fertilizing hermaphrodites, and a small proportion (<0.2%) being male (Hodgkin *et al.*, 1979). There are two other similarly androdioecious species in the genus, *C. briggsae* (*Cbr*) and *C. tropicalis* (*Ctr*). All three species evolved their hermaphroditism separately and uniquely (Ellis and Lin, 2014). Of the male-attracting ascarosides secreted by *C. elegans* (ascr#2, ascr#3, ascr#4, and ascr#8), ascr#8 is the most potent (Pungaliya *et al.*, 2009). Since ascr#8 is a male attractant in this hermaphroditic species, we asked if other hermaphroditic species retained the ability to attract males using this cue. Males from the gonochoristic (male-female) sister species to *C. briggsae* and *C. tropicalis – C. nigoni* (*Cni*) and *C. wallacei* (*Cwa*), respectively – were also assayed for their ability to respond to ascr#8. The closest relative of *C. elegans*, the gonochoristic *C. inopinata* (*Cin*, formerly *C. sp. 34*), which has been recently characterized (Kanzaki *et al.*, 2018), was also tested, along with the *Japonica* Group gonochoristic species *C. japonica* (*Cja*) and *C. afra* (*Caf*).

Dwell times were analyzed as previously described using a Spot Retention Assay (Narayan *et al.*, 2016). Dwell times were transformed using a Base 2 Exponentiation (2*^n^*, wherein *n* is equal to the raw dwell time value) to generate only non-zero data in order to calculate fold-changes. The Logbase2 of the fold-changes was then calculated to normalize the data. All data sets were first checked for normality using a D’Agostino & Pearson normality test before comparisons were performed. Species in which both vehicle and ascr#8 dwell times were normally distributed were compared using a paired *t*-test, while those in which one or both data sets were not normally distributed were compared using a Wilcoxon matched-pairs signed rank test (GraphPad Prism 7.03). Transformed values were compared between related species via an unpaired *t*-test.

*C. elegans* responded strongly to ascr#8, supporting our previously published results using the Spot Retention Assay (Pungaliya *et al.*, 2009; Narayan *et al.*, 2016). The other well studied hermaphroditic model species, *C. briggsae*, responded significantly, although not robustly as *C. elegans*, spending approximately 40 seconds in ascr#8 compared to *C. elegans*’ dwell time of nearly 150 seconds. (Fig. 1A). Surprisingly, the sister-species of *C. briggsae*, *C. nigoni*, responded in a more robust manner (Fig. 1A, B). Similarly, while *C. tropicalis* exhibited no response to ascr#8 – its dwell time in ascr#8 being no different than that of the vehicle control (Fig. 1A) – *C. wallacei* spent significantly more time in ascr#8 than the vehicle (Fig. 1A), and therefore exhibited significantly more attraction than *C. tropicalis* (Fig. 1B). These data suggest that females of gonochoristic species have retained their ability to attract males via ascr#8 signaling, while in androdioecious species, this communicatory mechanism is lost, potentially due to a lack of selective evolutionary pressure. However, neither *Japonica* Group member responded to ascr#8, nor did the closest *C. elegans* relative, *C. inopinata* (Fig. 1A). This lack of attraction to ascr#8 may be due to the highly specialized insect-commensal life-cycle of *C. inopinata*. This species may have lost the necessary olfactory receptors to sense ascr#8 within its reduced genome (Kanzaki *et al.*, 2018), in a manner similar to *Trichinella* (Srinivasan *et al.*, 2013).

Given that hermaphroditism evolved multiple times during nematode evolution, it is plausible that *C.briggsae* and *C. nigoni* both inherited mechanisms to sense ascr#8. However, given the recent evolution of *C. briggsae* (between 100,000 to 1 million years ago) (Cutter *et al.*, 2010; Thomas *et al.*, 2015), the reduced response compared to *C. nigoni* indicates less selective pressure to retain ascr#8 sensing mechanisms. Similarly, *C. tropicalis* likely evolved the ability to self-fertilize even before the *nigoni-briggsae* split (Stevens *et al.*, 2019), leading to its inability to respond attractively to ascr#8. Together, these data suggest that ascr#8 functions as a *C. elegans* species-specific male attractant, with other species having lost the necessary mechanisms to sense and respond to this chemical. As different neurons and receptors may play roles in the ascr#8-mediated dauer development pathways, the comprehensive ability to sense this pheromone in the species tested is yet to be determined.

## Reagents

The *C. elegans* strain CB1489 (*him-8(e1489)*) was obtained from Maureen Barr at Rutgers University. RE980 (*C. briggsae him-8(v188)*) and RE1017 (*C. tropicalis (him-8(v287)*) were generously provided by Ronald Ellis at Rowan University. The *C. wallacei* (JU1873), *C. nigoni* (JU1422), *C. afra* (JU1286), and *C. japonica* (DR5081) wild isolates were obtained from the *Caenorhabditis* Genetics Center. *C. inopinata* (NK74SC) was generously provided by the Forestry and Forest Products Research Institute in Japan.
